# Nonlinear convection stagnation point flow of Oldroyd-B nanofluid with non-Fourier heat and non-Fick’s mass flux over a spinning sphere

**DOI:** 10.1038/s41598-024-51475-z

**Published:** 2024-01-08

**Authors:** Gadisa Kenea, Wubshet Ibrahim

**Affiliations:** Department of Mathematics, Ambo University, Ambo, Ethiopia

**Keywords:** Mathematics and computing, Nanoscience and technology

## Abstract

The current paper concerned with a non-linear convection flow of the Oldroyd-B nanofluid at a point of stagnation across a rotating sphere under the influence of convective heat and passive control conditions. The analysis of energy and concentration transition has been scrutinized based on the Cattaneo–Christov diffusion model. The formulated coupled mathematical problem involving boundary requirements can be alerted to a set of highly nonlinear ordinary differential equations by employing similarity analysis. The numerical solution for the governing problem was computed by utilizing bvp4c solver method. The performance of velocity fields, skin friction drag, energy, heat transfer rate, and concentration for various control parameters has been analyzed using diagrams and tables. The findings stipulated that velocity, temperature, and nanoparticle are enhanced for the relaxation time constant while they decay for the retardation time parameter. The upshots also confirmed that enlarging magnetic parameters leads to improve both linear velocity and coefficient of skin friction. The velocity profiles are enhanced as a function of the rotation constant. But, normal velocity declines for buoyancy force ratio, and the same trend is being noted for magnetic and relaxation time parameters on angular velocity. The fluid temperature declines for the Prandtl number and augments for thermal convective parameter. The coefficient of skin friction decreases for larger thermal relaxation and rotation parameters, whereas an analogous effect is being noticed for Brownian parameter on the concentration field. Further, the thermophoresis parameter displays an enhancing tendency on temperature as well as concentration profile while bringing down the Nusselt number. The Lewis number and solutal relaxation parameter filter the concentration field. The graph of the streamline is sketched for identical values of the magnetic parameter and noticed that the contour lines increased as magnified. Confirmation of the current outcomes with former studies is presented.

## Introduction

The analysis of heat transfer in the non-Newtonian fluids are more complex than Newtonian fluid due to non-linear proportion existence in the slop velocity and tangential stress. Nowadays, a number of scholars are continuously working on the characteristics of non-Newtonian liquids in individual aspects, due to they acquired a huge interest in industrial, commercial and engineering applications, for example food processing, chemical reactions, material handling, oil storage and so on. So, in light of the peculiarities of nonlinear substances, many nonlinear kinds of models were offered. Sarpkaya^1^ studied first steady state non-Newtonian fluid transport in the presence of uniform magnetic parameter effects. Oldroyd B fluid is one of the non-Newtonian model used to describe the flow of viscoelastic fluids that establishes the characteristics of both relaxation and retardation times, which is introduced by Oldroyd^2^. This model can be regarded as an extension of the upper-convected Maxwell model and equivalent to a fluid filled with elastic bead and spring dumbbells. The importance of Oldroyd-B fluid in fluid mechanics lies in its ability to predict the complex flow phenomenon involving the behavior of dilute polymer solutions^3^.

Choi and Eastman^4^ floated the novel concept of fluid, which are interrupted by metallic or non-metallic nanomaterials known as nanofliuds. This simple derivation of Choi model concerned in the increment of heat transmission just for the greater thermal conductivity. Following this effective endeavor, a number of empirical frameworks have been developed and many studies are conducted to look into the synthesis of nanofluids and the effect of thermophysical features on different flow issues. Khan and Gorla^5^ discussed the movement of energy and mass of the non-Newtonian nanofluid flow through a non-isothermal sheet. Nadeem et al.^6^ initiated the magnetohydrodynamic flow of heat transmission on the Maxwell fluid around a stretching surface with nanoparticles effect. The analysis of magnetohydrodynamic flow in the Casson nanofluid through a porous shrinking sheet under the effects of convective condition and suction/injection is presented by Haq et al.^7^. Further, Azeem Khan et al.^8^ scrutinized the steady bidirectional free convective flow of Oldroyd-B nanofluid across an elongating surface. Shehzad et al.^9^ have presented the influences of convective conditions in the motion of magnetohydrodynamic nanofluid through an elastic plate. Hayat et al.^10^ investigated an electrically conducting MHD flow of couple stress nanofluid past stretching sheet with the existence of nanoparticle effects. Additionally, Hayat et al.^11^ Analyzed the motion of MHD in the Oldroyd B nanoliquid with convective heat transmission induced by an extending surface under the appearance Brownian motion and the thermophoresis effect. Consequently, Ahmad et al.^12^ explored unstable bidirectional movement on the Maxwell nanofluid in a permeable media with the features of Brownian and thermophoresis over stretching sheet. The motion of an electrically conducting MHD convinced due to a bidirectional Oldroyd-B nanofluid across a spreading surface was analyzed by Gupta and Gupta^13^. Also, radiative flow of magnetic Oldroyd-B nanofluid subjected to thermal and mass stratification with heat source/sink towards stretching cylinder scrutinized by^14,15^.

It is impossible to ignore the challenges of temperature and concentration differences near and distant from the wall owing to the demands of a substantial disparity between them. As a result, both the chemical and physical features of the flow field evolved as a means to adapt to the advanced technology utilized in diverse industrial manufacturing procedures. On such occasions, the inquiry of nonlinearity ought to be included to assist changes in interaction between temperature, density and concentration. Which prompts experts to find out the implications of thermo-physical features regarding the flow system. Among those, Sinha^16^ analyzed normal density temperature differences on the boundary layer flow of free convective under the consideration of frictional heating effects. Vajravelu and Sastri^17^ have studied the flow between two parallel plates due to the variation of quadratic density with temperature and found that it considerably affects the flow and heat transfer rates.

Further, nonlinear convection has a significant impact on a number of manufacturing industries, particularly heat pumps, the combustion process, geothermal energy sources, the production of natural gas, refrigeration of electrical factors, medicinal products, etc. Due to this reason, Kameswaran et al.^18^ examined the boundary layer flow with the influence of nonlinear convection and thermophoresis in a permeable medium across a perpendicular wall. Hayat et al.^19^ investigated the system of three dimensional Sisko nanofluid flow with combined convective over an elastic surface in the thermal and solutal convective circumstance. Nonlinear combined convection flow on the tangent hyperbolic fluid with nanoparticles through enlarging plate in the company of entropy generation has been studied by Khan et al.^20^. It is disclosed that the velocity of the fluid constantly rises with the nonlinear convection parameter. Hayat et al.^21^ investigated the motion of combined quadratic convection on the Maxwell nanofluid in the electrically conducting flow mechanism around a stretching cylinder under convective surface condition. Irfan, Khan, Gulzar and Khan^22^ investigated mixed convection flow of Oldroyd-B nanofluid with the impact of a stretching cylinder. The system of electrically conducting motion through nonlinear convective nanofluid flow under the existence of joule heat and thermal radiation was presented by Uddin et al.^23^. Further, Patil and Kulkarni^24^ discussed the flow of magnetohydrodynamic nanofluid in combined convection under the impacts of an irregular plane through a moving sheet. In addition, Patil et al.^25^ have investigated the tripled diffusive flux for mixed quadratic convection nanofluid with viscous dissolution effects around a wedge in the presence of convective heat constraints. Irfan^26^ studied nonlinear mixed convective flow of MHD Carreau nanofluid under variable properties.

The analysis of mass and energy transfer phenomena has acquired a huge interest for many researchers because of its numerous uses in manufacturing, commercial, and technological areas. Fourier^27^ for the first time coined the most effective model known as heat flux model with definite barriers to realize the system of heat transport in different circumstances. Cattaneo^28^ also offered a constructive modification of the Fourier model by adding the required value for the heat relaxation term as a means to address this challenge. This change is hyperbolic energy equation and accommodate the movement of heat through cultivation of thermal waves having restricted speed. subsequently, Christov^29^ added time product in the Maxwell–Cattaneo theory called Cattaneo–Christov heat flux model a view to conserve the material-constant formulation. Hayat et al.^30^ probed movement of heat transport in an electrically conducting flow of Oldroyd B fluid with magnetic field effects through an infinite sheet. Ciarletta and Straughan^31^ examined the uniqueness and physical stability of the Christov model. The boundary layer flow of coupled and energy transfer in the upper convected Maxwell fluid with the consideration of heat and mass flux model were studied by Han et al.^32^. The system of heat and mass conduction on the viscoelastic non-newtonian fluid flow with nanoparticles across an expanding sheet has been investigated by^33–35^. In addition, Irfan, Khan and Khan^36^ examined the non-Fourier heat flux and chemical reactions effect on the Oldroyd-B fluid flow under variable conductivity across a stretched cylinder. Khan and Nadeem^37^ analyzed the effect of external magnetic field and viscous dissipation on the chemically reactive flow of unsteady bio-convective Maxwell nanofluid across an exponentially stretching sheet subjected to variable slip boundary constraints. The behavior of variable thermal transportation in the micropolar fluid flow utilizing Fourier’s heat flux model with the existence of energy generation has been scrutinized by Khan, Nadeem and Muhammad^38^. Further, Ahmad, Nadeem, Muhammad and Khan^39^ examined mixed convection flow in the micropolar nanofluid under Cattaneo–Christov heat flux with variable thermal relaxation time due to stretching surface. Recently, Khan et al.^40^ explored the performance of heat and mass transition with modified heat flux theory and surface-catalyzed reactions within Sutterby ternary-hybrid nanofluid flow under the influence of partial slip velocity and convective boundary conditions.

Having an empirical basis in geophysics, fluid motion within a rotating structure finds extensive industrial use in mechanical sorting procedures, turning constructions, and food processing. Wang^41^ first involved the study of two dimensional fluid flow within the rotating system. Also, he presented a factor which contains the relationship proportion between rotation and stretching rate. The time dependent boundary layer flow with a permeable medium in a spinning fluid due to the instantly a moving sheet has been studied by Nazar et al.^42^. Additionally, Chamkha and Ahmed^43^ Investigated the mechanism of mass and energy transmit with mixed convection on the viscous MHD fluid over spinning sphere including heat generation and chemical reactions. Also, Khan et al.^44^ analyzed system of energy and mass transport applying Cattaneo–Christove heat and mass conduction model on the bidirectional Oldroyd B fluid through expanding surface. Then after, Hayat et al.^45^ described the steady electrically conducting magnetohydrodynamic flow of viscous nanofluid under velocity slip effect around a rotating disk. The features of momentum and energy transport in the MHD flow of a Jeffrey, Maxwell, and Oldroyd-B nanofluids with the existence of uniform magnetic field and radiation effects has been scrutinized by Sandeep and Sulochana^46^. Ibrahim et al.^47^ investigated the system of heat and mass conduction with mixed convection flow on the Oldroyd-B nanofluid over an expanding sheet under higher order slip, convective heat, and zero mass flux conditions.

Mahdy et al.^48^ examined the magnetic mixed convection flow with entropy generation analysis on the Casson nanofluid across rotating sphere including convective heat surface. Accordingly, the flow of magnetized Oldroyd-B fluid over a rotating disk with the impact of thermal radiation is discussed by Khan et al.^49^. Moreover, Hafeez et al.^50^ studied the movement of heat and mass by employing Cattaneo–Christove model on the viscoelastic Oldroyd B nanoliquid through a rotating flow in the presence of heat joule. Khan and Nadeem^51^ analyzed the impact of thermophoretic and thermal conductivity on the revolving Maxwell nanofluid flow with double stratification through linear/exponential stretching surface. Ibrahim et al.^52^ presented a communication report over viscoelastic nanofluid with Christov heat conduction theory in a bidirectional stretching sheet considering convective surface and the slip conditions. The study of chemical reaction with radiated flow on the Oldroyd-B nanofluid subjected with heat-mass convective condition were investigated in^53,54^. Furthermore, Khan et al.^55^ studied the temperature-dependent fluid property with chemical reaction on an electrically conducting flow of micropolar fluid between two parallel sheet accompanied by diffusion theory and slip effect in a rotating system.

As per prior literature, the nonlinear mixed convection on the Oldroyd-B nanofluid flow within the stagnation point about a rotating sphere has not been studied yet. In light of this, the intent of the current study is to analyze the nonlinear combined convection MHD flow on the Oldroyd-B nanofluid around a spinning sphere within the forward stagnation point under thermal convection and zero mass flux circumstances. Further, non-Fourier heat and non-Fick’s mass flux conditions are incorporated for the analysis of heat and mass transport phenomena. The formulated mathematical equations are transformed into their corresponding higher order ODE’s using the proper non-dimensional analysis, which is then numerically solved through bvp4c solver method. Additionally, the numerical solution is analyzed and discussed using figures and tables for distinct pertinent control variables by utilizing MATLABR2023a software. The reality of the solution confirms the remarkable result via comparison with some already-existing literature.

## Mathematical problem

**Figure 1:**
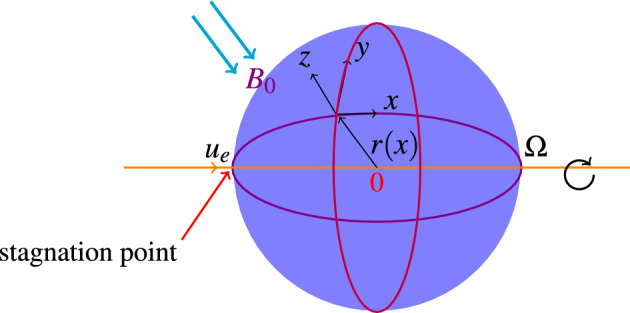
Geometrical flow model.

Consider steady mixed nonlinear convection flow of the Oldroyd-B nanofluid around a spinning sphere with convective heat conditions in the forward stagnation point region. Second order slip and no mass flux conditions were applied for velocity and concentration respectively. Further, Cattaneo–Christov conduction model was utilized to generate the governing equations for energy and concentration. The sphere revolving with invariant angular velocity $\Omega$ through an axis parallel to the ambient flow direction. The radius of a portion orthogonal to the sphere’s axis of length x along a culmination measured from the origin is considered as *r*(*x*). A physical flow model along with geometrical structure is depicted in Fig. 1.

i. The distance measured from the forward stagnation point on the surface of sphere is *x*
ii. The length in the axis of rotation is measured by *y* and the length against the surface of sphere normal to *x* and *y* axes is *z*.

Based on the above hypothesis, the mathematical formulation that governs the flow of boundary layer analysis stated in^48,50^:

1 $$\begin{aligned}{} & {} \frac{\partial (ru)}{\partial x} + \frac{\partial (rw)}{\partial z} = 0, \end{aligned}$$

2 $$\begin{aligned}{} & {} u\frac{\partial u}{\partial x} + w \frac{\partial u}{\partial z} - \frac{v^2}{r}\frac{dr}{dx} = \upsilon \frac{\partial ^2 u}{\partial z^2}-\frac{\sigma }{\rho }B_0^{2}(u + \lambda _1 w\frac{\partial u}{\partial z} - u_e) \\{} & {} \quad + u_e\frac{\partial u_e}{\partial x} - \lambda _{1} \left( u^2 \frac{\partial ^2 u}{\partial x^2} + w^2 \frac{\partial ^2 u}{\partial z^2} + 2uw \frac{\partial ^2 u}{\partial x \partial z} - \frac{2uv}{r} \frac{\partial v}{\partial x} - \frac{2vw}{r} \frac{\partial v}{\partial z} + \frac{uv^2}{r^2} + \frac{v^2}{r}\frac{\partial u}{\partial x}\right) \\{} & {} \quad + \upsilon \lambda _{2} \left( u \frac{\partial ^3 u}{\partial x \partial z^2} + w \frac{\partial ^3 u}{\partial z^3} - \frac{1}{r} \left(\frac{\partial u}{\partial z} \right)^2 - \frac{\partial u}{\partial x} \frac{\partial ^{2} u}{\partial z^{2}} - \frac{\partial u}{\partial z} \frac{\partial ^2 u}{\partial x \partial z} - 2\frac{\partial u}{\partial z} \frac{\partial ^2 w}{\partial z^2} \right) \\{} & {} \quad + g[\alpha _{1}(T-T_{\infty }) + \alpha _{t}(T-T_{\infty })^{2}]+ g[\alpha _{2}(C-C_{\infty }) + \alpha _{c}(C-C_{\infty })^{2}], \end{aligned}$$

3 $$\begin{aligned}{} & {} u\frac{\partial v}{\partial x} + w \frac{\partial v}{\partial z} + \frac{uv}{r} \frac{dr}{dx} = \upsilon \frac{\partial ^2 v}{\partial z^2}-\frac{\sigma }{\rho }B_0^{2}(v + \lambda _1 w\frac{\partial v}{\partial z}) \\{} & {} \quad - \lambda _{1} \left( u^2 \frac{\partial ^2 v}{\partial x^2} + w^2 \frac{\partial ^2 v}{\partial z^2} + 2uw \frac{\partial ^2 v}{\partial x \partial z} + 2\frac{uv}{r} \frac{\partial u}{\partial x} +2 \frac{vw}{r} \frac{\partial u}{\partial z} -2 \frac{u^2v}{r^2} - \frac{v^3}{r^2} + \frac{v^2}{r}\frac{\partial v}{\partial x}\right) \\{} & {} \quad + \upsilon \lambda _{2} \left( u \frac{\partial ^3 v}{\partial x \partial z^2} + w \frac{\partial ^3 v}{\partial z^3}-2 \frac{\partial v}{\partial z} \frac{\partial ^2 w}{\partial z^2} -\frac{1}{r}\frac{\partial u}{\partial z}\frac{\partial v}{\partial z}-\frac{\partial v}{\partial x}\frac{\partial ^2 u }{\partial z^2} + \frac{ v}{r}\frac{\partial ^2 u }{\partial z^2} - \frac{\partial v}{\partial z}\frac{\partial ^2 u }{\partial x \partial z} - \frac{u }{r}\frac{\partial ^2 v }{\partial z^2}\right) , \end{aligned}$$

4 $$\begin{aligned}{} & {} u \frac{\partial T}{\partial x} + w \frac{\partial T}{\partial z} + \tau _e \left( u^2 \frac{\partial ^2 T}{\partial x^2} + w^2 \frac{\partial ^2 T}{\partial z^2} + 2uw \frac{\partial ^2 T}{\partial x \partial z} + u \frac{\partial u}{\partial x} \frac{\partial T}{\partial x} + w \frac{\partial w}{\partial z} \frac{\partial T}{\partial z} + u \frac{\partial w}{\partial x} \frac{\partial T}{\partial z} + w \frac{\partial u}{\partial z} \frac{\partial T}{\partial x}\right) \\{} & {} \qquad = \alpha \frac{\partial }{\partial z} ( \frac{\partial T}{\partial z}) + \tau \left( D_{B}\frac{\partial C}{ \partial z} \frac{\partial T}{ \partial z} + \frac{ D_{T}}{T_{\infty }} (\frac{\partial T}{\partial z})^{2} \right) , \end{aligned}$$

5 $$\begin{aligned}{} & {} u \frac{\partial C}{\partial x} + w \frac{\partial C}{\partial z} + \tau _c \left( u^2 \frac{\partial ^2 C}{\partial x^2} + w^2 \frac{\partial ^2 C}{\partial z^2} + 2uw \frac{\partial ^2 C}{\partial x \partial z} + u \frac{\partial u}{\partial x} \frac{\partial C}{\partial x} + w \frac{\partial w}{\partial z} \frac{\partial C}{\partial z} + u \frac{\partial w}{\partial x} \frac{\partial C}{\partial z} + w \frac{\partial u}{\partial z} \frac{\partial C}{\partial x}\right) \\{} & {} \quad = D_{B} \frac{\partial ^2 C}{\partial z^2} + \frac{D_{T}}{T_{\infty }}\frac{\partial ^2 T}{\partial z^2}, \end{aligned}$$

Subject to the proper boundary conditions,

6 $$\begin{aligned}{} & {} u = U_{w}(x)+ U_{slip}, v = \Omega x, \quad w = 0, -k \frac{\partial T}{\partial z} = h_f(T_{f} - T), \quad D_{B} \frac{\partial C}{\partial z} + \frac{D_{T}}{T_{\infty }}\frac{\partial T}{\partial z} = 0, \quad at \quad z = 0, \\{} & {} \quad u \rightarrow u_{e}, \quad v \rightarrow 0, \quad T \rightarrow T_{\infty }, \quad C \rightarrow C_{\infty }, \quad as \quad z \rightarrow \infty . \end{aligned}$$

where u, v, w the velocities in the *x*, *y*, and *z* directions , respectively. Further, $\mu$ is the absolute viscosity, $\rho$ is fluid density, $\upsilon = \frac{\mu }{\rho }$ is fluid resistance, $\lambda _1$ and $\lambda _1$ the relaxation and retardation times, $\tau = (\rho c_p ) /(\rho c_p)_f$ is heat capacity ratio between nanoparticle to base fluid, $c_{p}$ specific heat, $\kappa$ is the vortex viscosity, T stands for temperature, K is the thermal conductivity, g represents gravity, $D_{B}$ the Brownian diffusion coefficient, $D_{T}$ stands for the thermophoretic diffusion coefficient, $\sigma$ is electrical conductivity, and $B_{0}$ represents magnetic field. Consider the given similarity variables^44,48^

7 $$\begin{aligned} \eta & = \sqrt{\frac{a}{\upsilon }}z, \quad u = ax f'(\eta ), \quad v = \Omega xg(\eta ), \quad w = -(a \nu )^{1/2}f(\eta ), \\ \theta (\eta ) & = \frac{T - T_\infty }{T_{f} - T_{\infty }}, \quad \phi (\eta ) = \frac{C - C_{\infty }}{C_{w} - C_{\infty }}, \end{aligned}$$

By using similarity variables of Eq. (7), the transport equation Eq. (1) is fulfilled. Hence, Eqs. (2)−(5) ) subsequently transformed into the set of nonlinear standard equations.

8 $$\begin{aligned}{} & {} f'''+ ff''- f'^2 + \lambda (g^2 -\beta _1 g^2f' - 2\beta _1 fgg') + M ( 1 + \beta _1ff''-f') + 1 + \beta _1 \left( 2ff'f'' - f^2f''' \right) \\{} & {} \quad - \beta _2 ff'''' + (1 + \lambda _t \theta )\theta + N(1 + \lambda _s \phi )\phi = 0, \end{aligned}$$

9 $$\begin{aligned}{} & {} g'' + fg'+ M(\beta _1 fg' - g) + \beta _1(2ff'g' + gff'' - fg'' - gf'^2) \\{} & {} \quad + \beta _2 (f'g''-fg''' + gf'''-\sqrt{\lambda } gg''-fg'') = 0, \end{aligned}$$

10 $$\begin{aligned}{} & {} \frac{1}{Pr}\theta '' + f \theta ' - \delta _t \left( f^2\theta '' + ff'\theta ' \right) + Nt\theta '^{2} + Nb \phi ' \theta ' = 0, \end{aligned}$$

11 $$\begin{aligned}{} & {} \phi '' + PrLe \left( f\phi ' - \delta _c f^2 \phi ''\right) - PrLe\delta _c ff' \phi ' + \frac{Nt}{Nb} \theta '' = 0, \end{aligned}$$

With the transformed boundary circumstances,

12 $$\begin{aligned}f(0) &= 0, \quad f'(0) = \gamma f''(0) + \delta f^{\prime\prime\prime}(0), \quad g(0) = 1, \quad \theta (0) = \frac{1}{Bi}\theta '(0) + 1, \\ &\quad Nb \phi '(0) + Nt \theta '(0) = 0, \quad {\text {at}} \quad \eta = 0 \\ &\quad f'(\eta ) \rightarrow 1, \quad g(\eta ) \rightarrow 0, \quad \theta (\eta ) \rightarrow 0, \quad \phi (\eta ) \rightarrow 0, \quad \text {for} \quad \eta \rightarrow \infty . \end{aligned}$$

Where *M*, $\beta _1$, $\beta _2$, $\lambda$ N, $G_r$, *Gc*, $\lambda _t$, $\lambda _c$ Pr, Le, $\delta _t$, $\delta _c$, Nb, Nt, $\gamma$, $\delta$, *Bi* are correspondingly denotes for magnetic field, relaxation and retardation time constant, rotation number, buoyancy force proportion, temperature Grashof number, concentration Grashof Number, thermal and concentration nonlinear convection variables, Prandtl number, Lewis number, relaxation times parameters for temperature, relaxation times parameters for concentration, Brownian movement, thermophoresis number, first and second order slip parameter, Biot number and this involved parameters are defined by:

$$\begin{aligned}M& = \frac{\sigma }{\rho a}B_0^2, \quad \beta _1 = \lambda _1a, \quad \beta _2 = \lambda _2a, \quad N = \frac{Gc}{Gr} = \frac{\alpha _2 (C_w-C_{\infty} )}{\alpha _1(T_f-T_{\infty} )}, \quad Gr = \frac{a^2g \alpha _1(T_f - T_{\infty} )}{\upsilon ^2},\\ &Gc = \frac{a^2g\alpha _2(C_{w}-C_{\infty })}{\upsilon ^2}, \lambda = \left( \frac{\Omega }{a}\right) ^2, \quad \lambda _t = \frac{\alpha _{t}}{\alpha _1}(T_f - T_{\infty} ), \quad \lambda _s = \frac{\alpha _{c}}{\alpha _{2}}(C_{w} - C_{\infty }), \quad Pr = \frac{\rho \upsilon c_{p} }{\kappa }, \\ &Le = \frac{\kappa }{D_B \rho c_p}, \delta _t= \tau _ea, \quad \delta _c = \tau _ca, \quad Nb = \tau \frac{D_B}{\upsilon }(C_{w} - C_{\infty }), \quad Nt = \tau \frac{D_T}{T_{\infty} \upsilon }(T_{\infty} - T_f), \\ &\gamma = A\sqrt{\frac{a}{\upsilon }}, \quad \delta = B\frac{a}{\upsilon }, \quad Bi = \frac{h_f}{\kappa }\sqrt{\frac{a}{\upsilon }} \end{aligned}$$

## Numerical procedure

The transformed nonlinear standard differential equations in Eqs. (8)–(11) subject to Eq. (12) can be minimized to linear system by employing bvp4c solver method. Then the numerical simulation has been developed in MATLAB program to obtain the solution with the relative error tolerance of $10^{-7}$. Here the following relations are introduced to reduce into first order:

13 $$\begin{aligned}f& = y_1, \quad f' = y_1' = y_2, \quad f'' = y_2' =y_3,\quad f'''=y_3' = y_4,\quad g = y_5,\quad g' = y_5' = y_6, \\ & g'' = y_6' = y_7, \theta = y_8, \quad \theta ' = y_8' = y_9, \quad \phi = y_{10}, \quad \phi ' = y_{10}' = y_{11}, \end{aligned}$$

Then, using Eq. (13) the system equations can be restated as:

$$\begin{aligned} \left( \begin{array}{c} y_1' \\ y_2' \\ y_3' \\ (\beta _2*y_2)* y_4' \\ y_5' \\ y_6' \\ (\beta _2*y_1)* y_7' \\ y_8' \\ y_9' \\ y_{10}' \\ y_{11}' \end{array} \right) = \left( \begin{array}{c} y_2, \\ y_3 ,\\ y_4, \\ y_4+ y_1*y_3- y_2^2 + \lambda (y_5^2 -\beta _1 y_5^2*y_2 - 2\beta _1 y_1*y_5*y_6)\\ + M ( 1 + \beta _1y_1*y_3-y_2) + 1 + \beta _1 \left( 2y_1*y_2*y_3 - y_1^2*y_4 \right) \\ + (1 + \lambda _t*y_8)*y_8 + N(1 + \lambda _s *y_10)*y_{10}, \\ y_6,\\ y_7,\\ \beta _1(2y_1*y_2*y_6 + y_1*y_3*y_5 - y_1*y_7 - y_5* y_2^2)\\ +y_7 + y_1*y_6 + M(\beta _1 y_1*y_6 - y_5)\\ + \beta _2 (y_2*y_7 + y_4*y_5-\sqrt{\lambda } y_5*y_7-y_1*y_7),\\ y_9, \\ \frac{-Pr*y_1*y_9 + Pr*\delta _t* y_1*y_2*y_9 - Pr*Nt*y_9^{2} - Pr*Nb*y_{11}*y_9}{1-Pr*\delta _t*y_1^2}, \\ y_{11}, \\ \frac{-Pr*Le*y_1*y_{11} + Pr*Le*\delta _c *y_1*y_2*y_{11}- \frac{Nt}{Nb}*y'_9}{1-Pr*Le*\delta _c * y_1^2}, \end{array}\right) \end{aligned}$$

The boundary conditions Eq. (12) in light of the new variables take the form:

$$\begin{aligned}&y_1(0) = 0, \quad y_2(0) = \gamma y_3(0) + \delta y_4(0), \quad y_5(0) = 1, \quad y_9(0) = -Bi(1-y_8(0)),\\&Nb y_{11}(0) + Nt y_9(0) = 0.\\&\quad y_2(\infty ) \rightarrow 1, \quad y_5(\infty ) \rightarrow 0, \quad y_8(\infty ) \rightarrow 0, \quad y_{10}(\infty ) \rightarrow 0. \end{aligned}$$

## Results and discussion

For the present investigation the numerical technique called bvp4c method were implemented to compute the system higher order differential equations Eqs. (8)–(11) with the corresponding limit conditions Eq. (12). The numerical solutions for velocities fields $f'(\eta )$ and $g(\eta )$, skin friction coefficient −$f''(0)$, energy $\theta (\eta )$, heat transfer rate −$\theta '(0)$, and concentration $\phi (\eta )$ distributions along various physical parameters were analyzed and displayed via figures and tables using MATLABR2023a software. Here the scale of control variables was defined in the following order based on history, existence, and uniqueness^44,45,56^, that is $0.1 \le \gamma \le 0.5$, $0.2 \le \delta \le 0.5$, $0.7 \le Pr \le 1.2$, $1 \le Le \le 3$, $0.1 \le M \le 1$, $0.0 \le \lambda \le 0.5$, $0.2 \le N \le 1$, $0.0 \le \beta _1 \le 0.5$, $0.1 \le \beta _2 \le 0.6$, $0.1 \le \lambda _t \le 0.5$, $0.1 \le \lambda _c \le 0.5$, $0.1 \le \delta _t \le 0.4$, $0.1 \le \delta _c \le 0.3$, $0.1 \le Bi \le 5$, $0.1 \le Nt \le 0.5$, $0.1 \le Nb \le 0.5$, while $\gamma = \delta = 0.5$, Le = B i = 2, M = 0.1, $\lambda = \beta _1 = \beta _2 = \delta _t = 0.1$, $\delta _c = 0.3$, Gc = Gr = 0.5, $\lambda _t = 0.2$, $\lambda _c = 0.1$, Nb = 0.5, and Nt = 0.2 are fixed values (unless stated). The solutions of this research achieved a remarkable acceptance with the earlier published data through comparison.

### Velocity profiles

**Figure 2:**
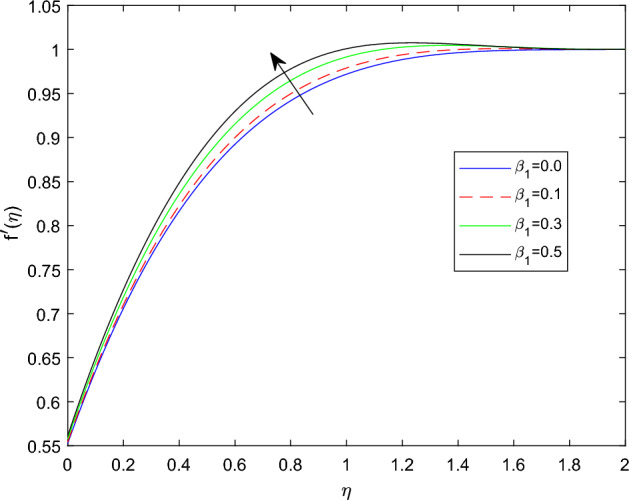
$f'(\eta )$ profile via $\beta _1$ for $Gr =\gamma = \delta = 0.25$ and Le = 1.

**Figure 3:**
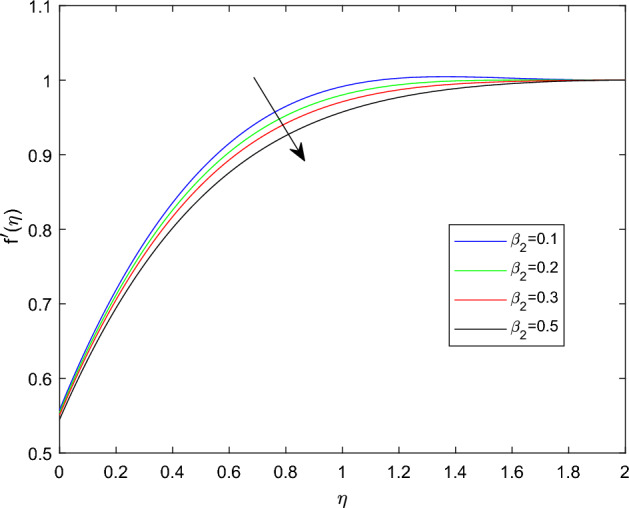
$f'(\eta )$ profile via $\beta _2$ for $\beta _1 =0.3$, $Gr =\gamma = \delta = 0.25$ and Le = 1.

**Figure 4:**
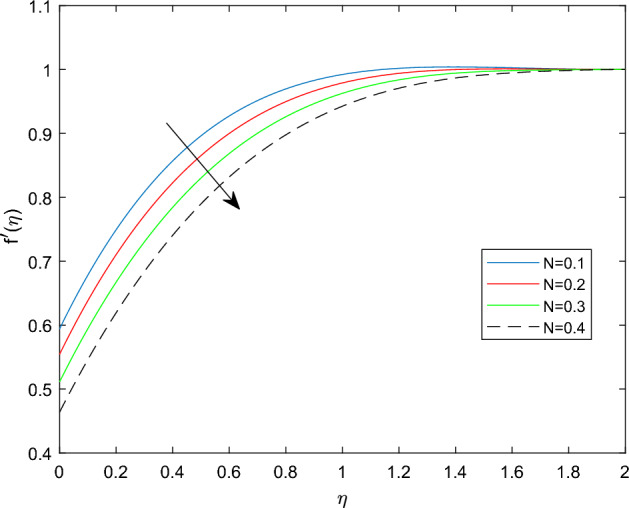
$f'(\eta )$ profile via N for $Gr =\gamma = \delta = 0.25$ and Le = 1.

Figures 2 and 3 sketched to demonstrate the influence of relaxation and retardation time variables towards linear velocity distributions $f'(\eta )$. They noted that enlarging the amount of relaxation time number $\beta _1$ leads to improve the velocity distribution, while the declining trend can be observed in velocity profile for large magnitude of retardation time parameter $\beta _2$. Figure 4 reveals that the characteristics linear velocity field $f'(\eta )$ for the augmenting ratios of buoyancy force N. The graph explored that for enhancing in the quantities of buoyancy force ratio the velocity field become degraded. This is because increasing in the buoyancy force ratio implies that there is larger concentration buoyancy force while lessen temperature buoyancy force, causing to a reduction in the convective currents and decline linear velocity field.

**Figure 5:**
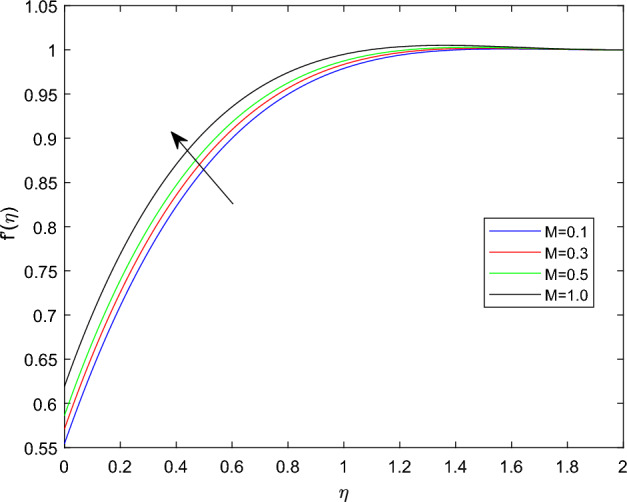
$f'(\eta )$ profile via M for $Gr =\gamma = \delta = 0.25$ and Le = 1.

**Figure 6:**
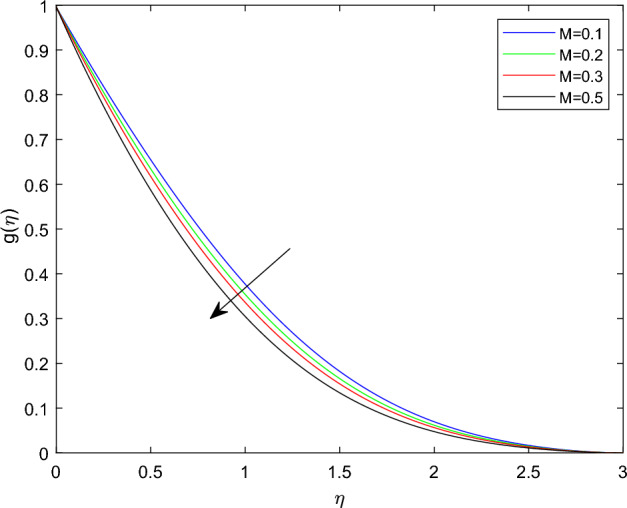
$g(\eta )$ profile via M for $\lambda _t = 0.5$, $\delta _c = 0.2$.

**Figure 7:**
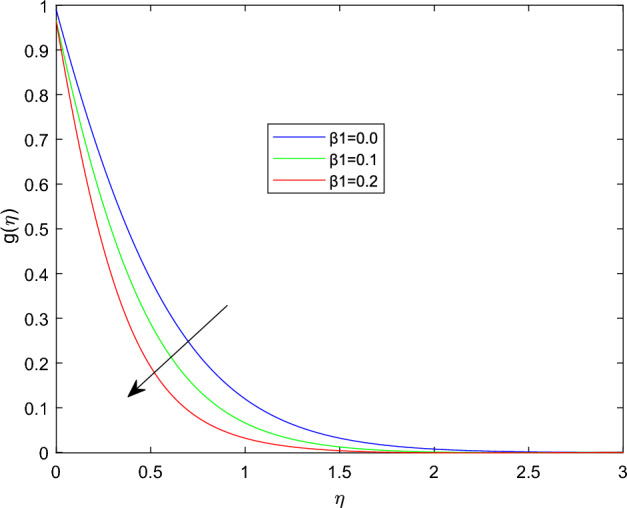
$g(\eta )$ profile via $\beta _1$ for M = Nt = 0.5.

**Figure 8:**
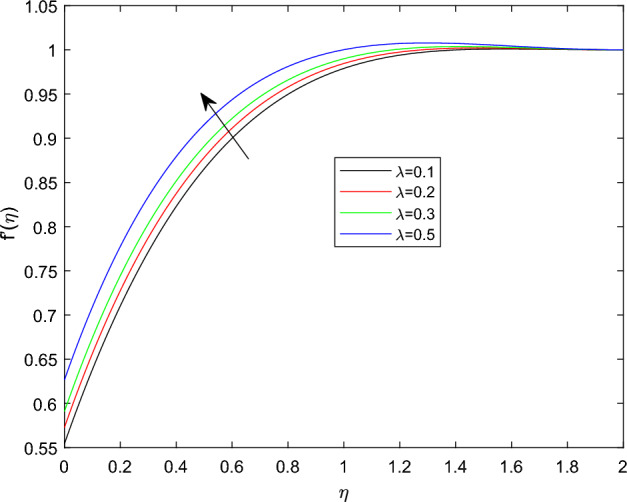
$f'(\eta )$ profile via $\lambda$ for $Gr =\gamma = \delta = 0.25$ and Le = 1.

**Figure 9:**
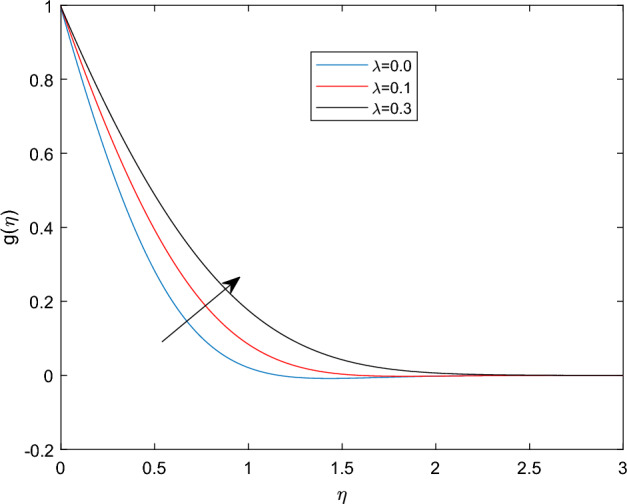
$g(\eta )$ profile via $\lambda$ for Le =1, $\beta _1 = Nt = 0.5$.

Figures 5 and 6 depicts the effect of magnetic field constant M along the components of fluid velocities $f'(\eta )$ and $g(\eta )$ accordingly. This indicates that normal velocity is boosting up as the numerical quantities of magnetic parameter M increase, whereas the angular velocity decline for large amount of M. This is due to the transverse magnetic field in the direction perpendicular to the flow field produces a resistant type force known as Lorentz force. Enhancing in the magnetic field strength causes to increase in the linear velocity of the particle due to the force acting on it increased. However, this can leads a decline in the angular velocity as the radius of the path rise due to strong magnetic field. The characteristics of rotational velocity $g(\eta )$ along the various magnitudes of relaxation time parameter $\beta _1$ is displayed through Fig. 7. That is clearly displayed from figure as the magnitudes of $\beta _1$ grows causes to enhancement in the angular velocity and thickness of its boundary. Figures 8 and 9 describe the variations of $f'(\eta )$ and $g(\eta )$ profiles together with the effect of rotation number $\lambda$. Which identifies that as the rotation parameter $\lambda$ growth the rotation become more concentrated around the axis, this means that the angular velocity increase and the linear velocity at any point on the object also increase.

### Temperature and concentration profiles

**Figure 10:**
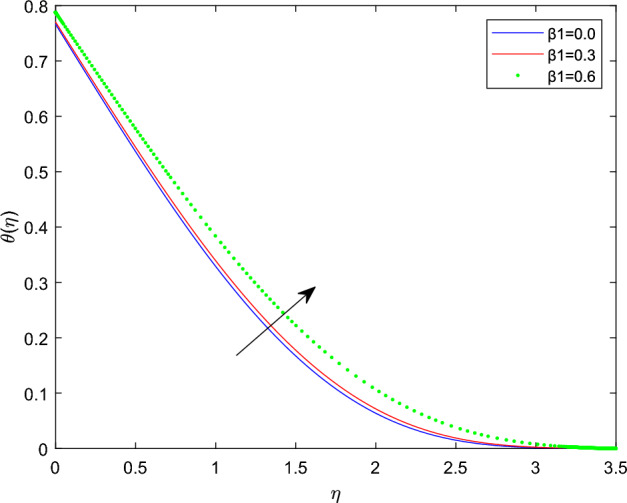
$\theta (\eta )$ profile via $\beta _1$ for M = $\lambda = Nt = 0.5$, $\gamma = \delta = \delta _c = 0.1$ $\beta _2 = 0.4$, and Bi = 5.

**Figure 11:**
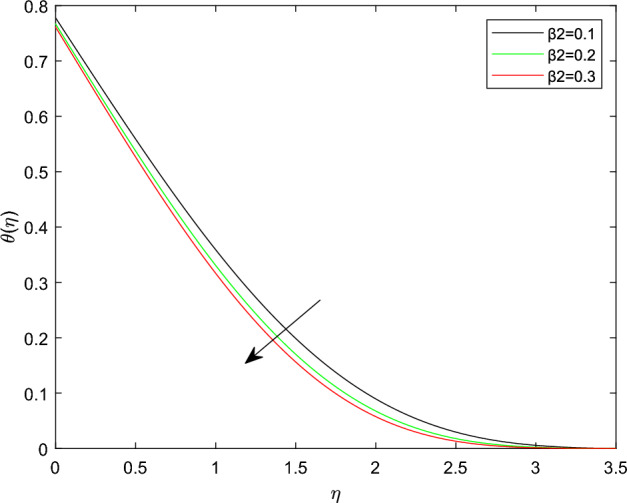
$\theta (\eta )$ profile via $\beta _2$ for $\gamma = \beta _1 = \delta = 0.1$, M = $\lambda = 0.5$, $\delta _c = 0.1$, and Nb = 0.3.

**Figure 12:**
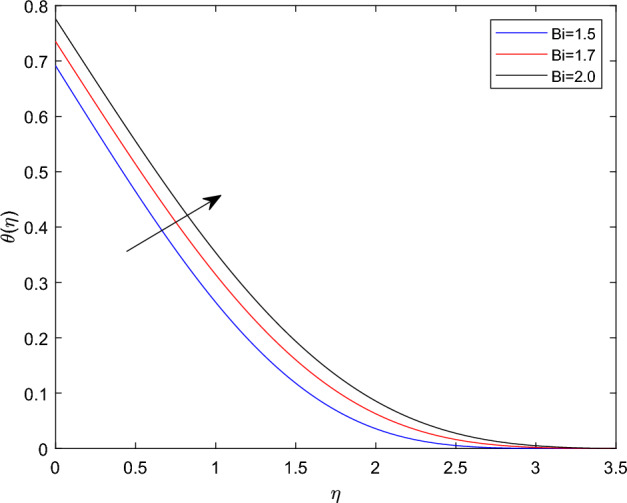
$\theta (\eta )$ profile via Bi for $\gamma = \delta = 0.1$, M = 0.5, $\delta _c = 0.1$ and $\lambda = 0.5$.

**Figure 13:**
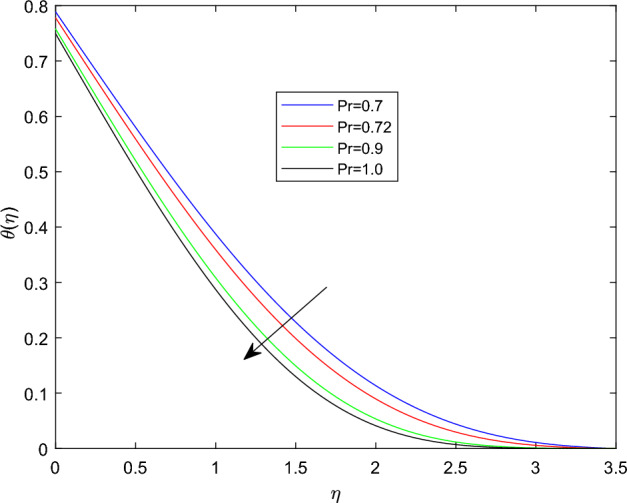
$\theta (\eta )$ profile via Pr for $\gamma = \delta = 0.1$, M = 0.5, Nb = 0.3, $\delta _c = 0.1$ and $\lambda = 0.5$.

**Figure 14:**
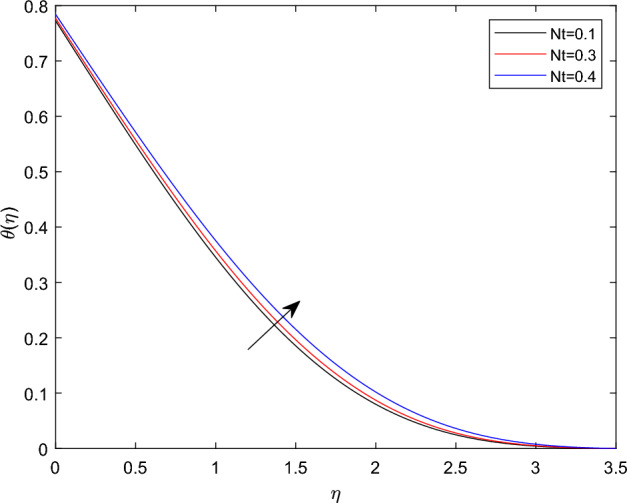
$\theta (\eta )$ profile via Nt for $\gamma = \delta = 0.1$, $\lambda _c = 0.2$, M = 0.5, Nb = 0.4, $\delta _c = 0.1$ and $\lambda = 0.5$.

Figures 10 and 11 plotted to explain how relaxation time parameter $\beta _1$ and retardation time parameter $\beta _2$ influence temperature distribution $\theta (\eta )$. From the two graphs the reverse characteristics are observed for enlarging values of $\beta _1$ and $\beta _2$ parameters. Generally, $\beta _1$ contains relaxation time, while $\beta _2$ involves retardation time. Therefore, an increment in $\beta _1$ related to high relaxation time which causes to create a force to resist the motion of fluid which strength temperature profile and for rise in retardation time constant $\beta _2$ corresponds to poor temperature profile. The behavior of thermal distributions $\theta (\eta )$ at distinct numerical quantities of Biot number Bi is outlined in Fig. 12. Which indicates that both energy and its thermal boundary enhances as the numerical quantities of Biot parameter Bi raises. It is owing to the fact that thermal resistance within the object is significant than convective resistance at the surface of sphere, which results in the increased of convective heat transmission of nanofluid and temperature profile. Figure 13 is plotted to portray the impression of Prandtl number Pr against temperature distribution $\theta (\eta )$ and layer of thermal boundary. That is, as Pr grows in magnitude the temperature distributions show decreasing behavior within the region. Physically, large amount of Pr stands for poor thermal diffusivity which prompts decrease temperature graph and thinner thermal boundary layer. Figure 14 illustrates that the variations of temperature distribution by considering various approximations of thermophoresis number Nt. It can be seen that both temperature profile and thickness of thermal boundary layer boosting up with the expansions of Nt. Physically, increasing in the values of Nt implies that more effect of the temperature gradient on the motion of particles causes to enhance the local temperature.

**Figure 15:**
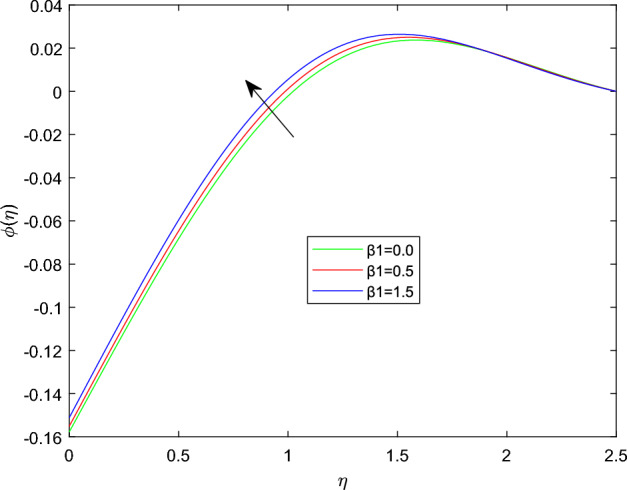
$\phi (\eta )$ profile via $\beta _1$ for Nb = 0.3, $\delta _c = \lambda _t = 0.1$, and $\lambda _c = 0.2$.

**Figure 16:**
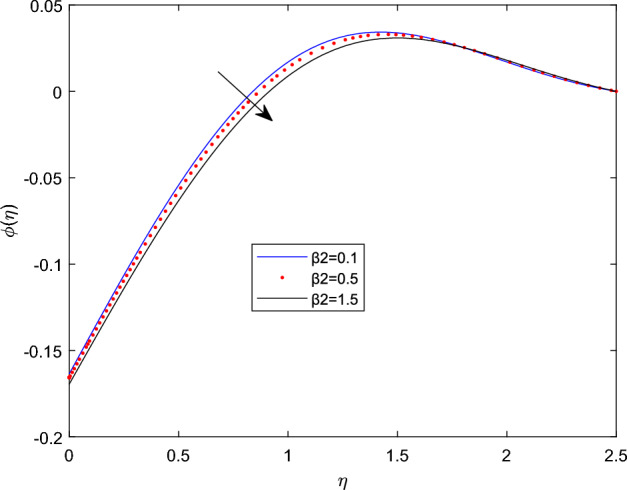
$\phi (\eta )$ profile via $\beta _2$ Nb = 0.3, $\delta _c = \lambda _t = 0.1$, and $\lambda _c = 0.2$.

**Figure 17:**
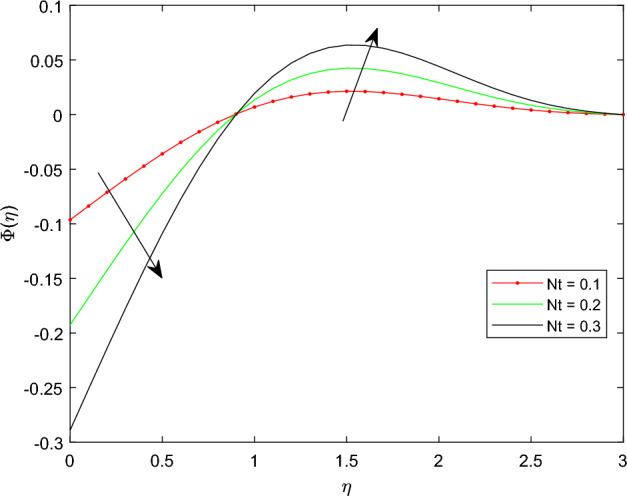
$\phi (\eta )$ profile via Nt Le = 0.3, Nb = 0.3, $\delta _c = \lambda _t = 0.1$, and $\lambda _c = 0.2$.

**Figure 18:**
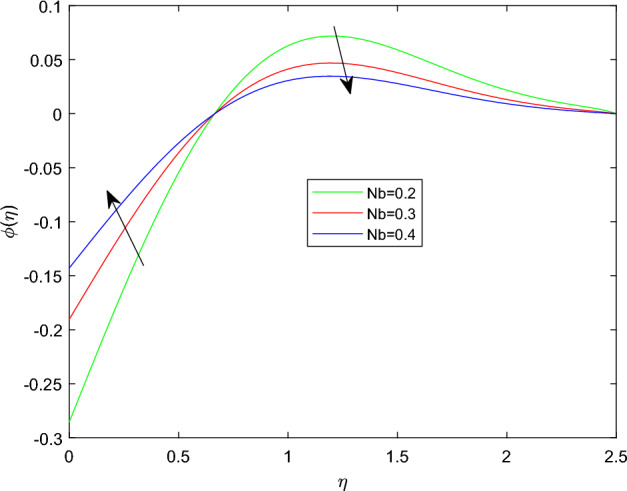
$\phi (\eta )$ profile via Nb for Le = 3, $\delta _c = \lambda _t = 0.1$, and $\lambda _c = 0.2$.

**Figure 19:**
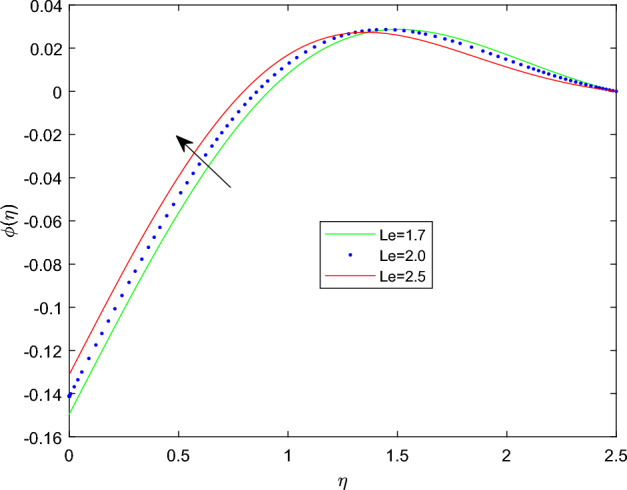
$\phi (\eta )$ profile via Le.

**Figure 20:**
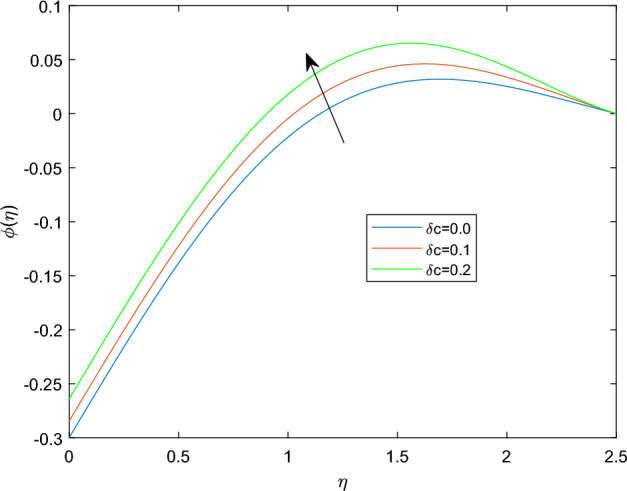
$$\phi (\eta )$$ profile via $$\delta _c$$.

Figures 15 and 16 are drawn to exhibit the property of nanoparticle dissemination $\phi (\eta )$ towards various estimations of relaxation time parameter $\beta _1$ and retardation time parameter $\beta _2$ respectively. Figure 15 indicated the spread of concentration and the boundary sheet improved for enlarging values of $\beta _1$. But for larger retardation time constant $\beta _2$, results a decay in nanoparticle dispersion and related bounding lines. Figures 17 and 18 display the change of nanoparticle distributions $\phi (\eta )$ with the alteration of thermophoresis Nt and Brownian number Nb. From figures it is interesting to see that both Nt and Nb have opposite effects near and far from the plane. Figure 17 depicts that the graph of concentration decreases near the surface and enhances far from the field via increasing amount of Nt. Due to the movement of fluid particles enhances for larger Nt that leads to less particles near the hot surface and they pronounced away from the plane, which owing to increase nanoparticle species. Figure 18 depicts that enlarging Nb gives the concentration profile $\phi (\eta )$ increases near the plane, but it diminishes away from the field. This is because increasing in Nb parameter means an increased intensity of random motion which causes more collisions and interactions between the particles, that can assemble near the surface. However as far from the surface the effect of Nb become smaller, results in a decline concentration profile. The response of the nanoparticle concentration corresponding to the estimation in the Lewis parameter Le and solutal relaxation period $\delta _c$ were figured in Figs. 19 and 20 respectively. It is found that as the values of Le surge up, the improvement trend is indicated in the nanoparticle concentration and the width of bounding line. Generally, larger Le indicates that bigger thermal diffusivity than mass diffusivity which results increase in the concentration profile. Similarly, Fig. 20 showed that the concentration profile enhanced as a function of $\delta _c$, because higher $\delta _c$ implies that slows down the diffusion of solute particles due to a concentration gradient.

**Figure 21:**
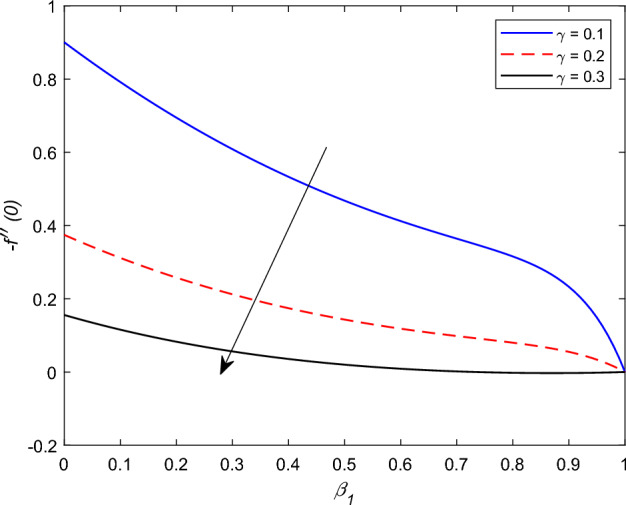
−$$f''(0)$$ profile via $$\gamma$$.

**Figure 22:**
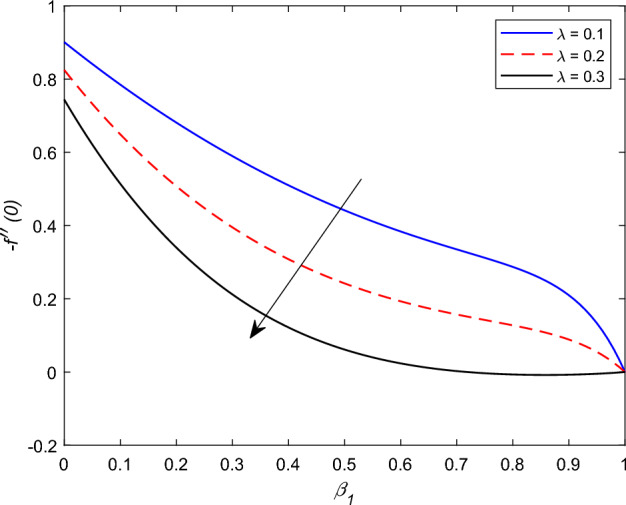
−$$f''(0)$$ profile via $$\lambda$$.

**Figure 23:**
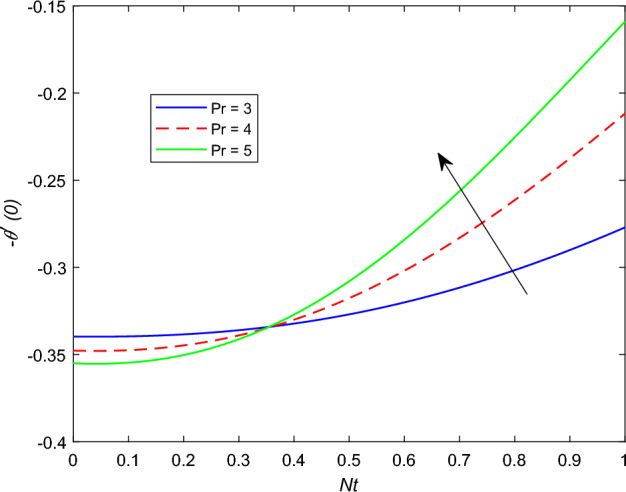
−$\theta (0)$ profile via Pr.

**Figure 24:**
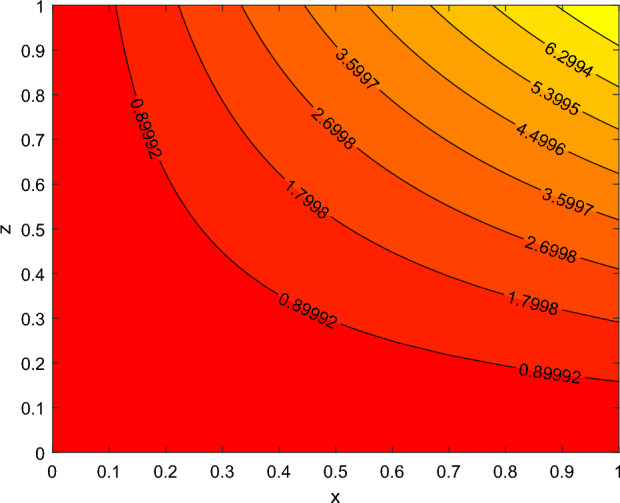
Streamlines for M = 0.0.

**Figure 25:**
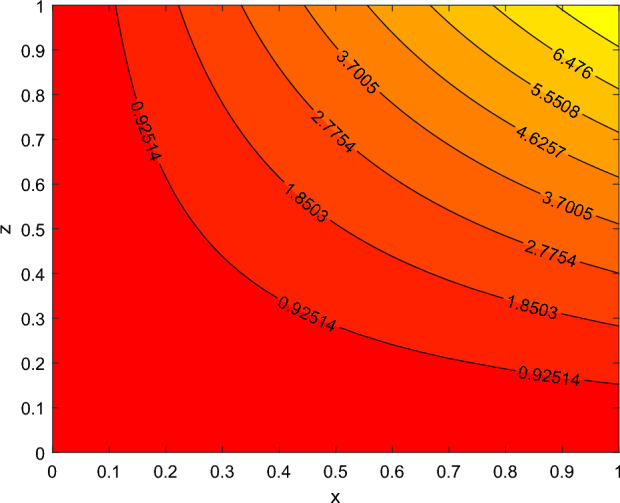
Streamlines for M = 0.5.

**Figure 26:**
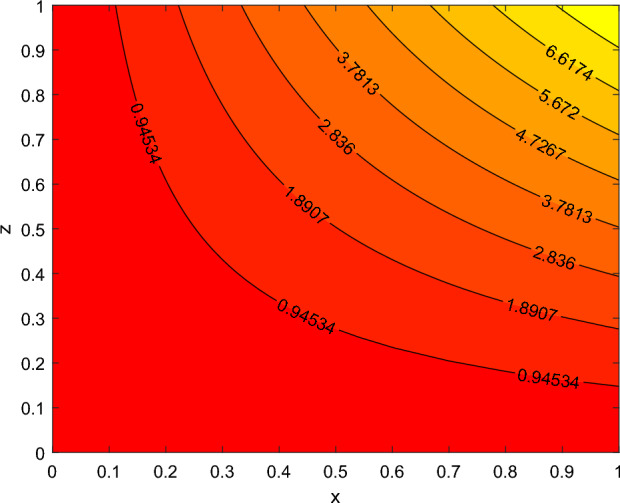
Stream lines for M = 1.

**Figure 27:**
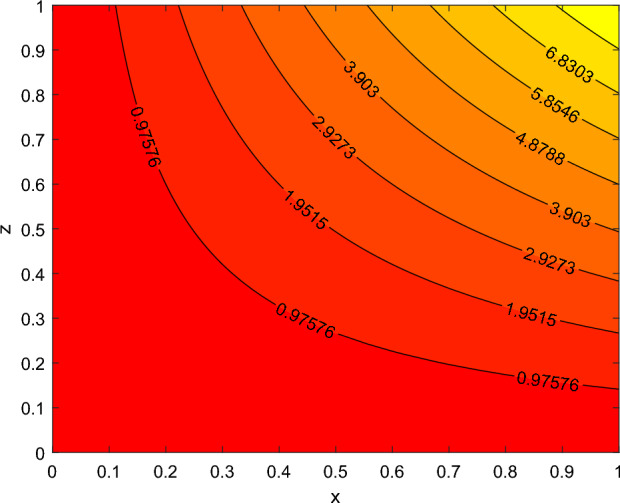
Streamlines for M = 2.

**Figure 28:**
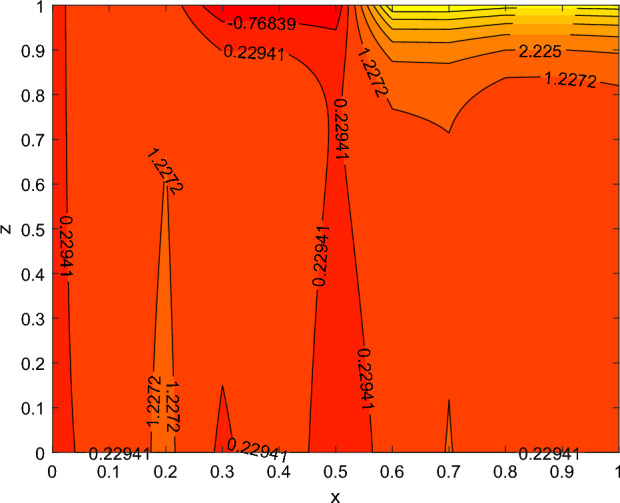
contour lines for M = 1.

**Figure 29:**
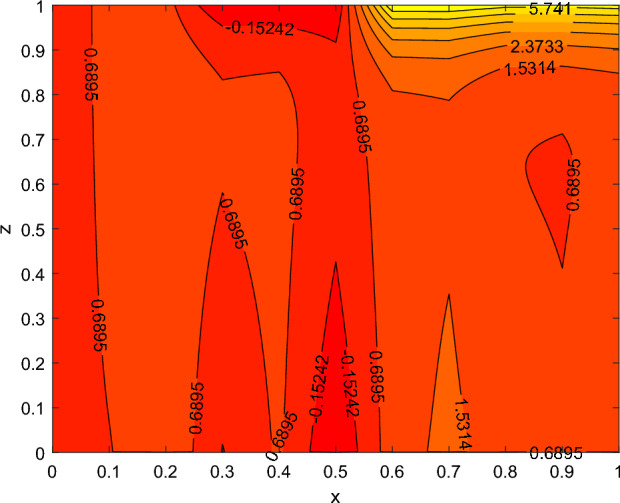
contour lines for M = 2.

The disparity in the coefficient of skin friction −$f''(0)$ and local heat transfer rate −$\theta '(0)$ under the influence of numerous pertinent material parameters has been explained through figures and tables below. Figure 21 described that the influence slip velocity constant $\gamma$ on the skin friction factor along relaxation time number $\beta _1$. The figure indicated that increment of $\gamma$ causes to decline −$f''(0)$ via relaxation time parameter $\beta _1$. Physically, when $\gamma$ enhances the fluid is allowed to slip more over the surface causes reduces the velocity gradient near the wall results in a reduced shear stress. Further, a decreasing tendency for −$f''(0)$ is observed via finer values of rotation parameter $\lambda$ alongside with $\beta _1$, as revealed in Fig. 22. Because mounting $\lambda$ values causes to down velocity gradient at the wall implies −$f''(0)$ is reduced. From Fig. 23 it is evidently noticed as the Prandtl number Pr rise up in magnitude besides thermophoresis number Nt leads to strength the local Nusselt number −$\theta '(0)$. Further, Figs. 24, 25, 26, 27 are portrayed to show the streamline graphs for distinct values of parameter M. They stated that increasing in the magnetic number, the graphs of streamline more presiding. Moreover, Figs. 28, 29 are pictured to display the curve of contour line for different values of magnetic parameter. The variation of physical quantities, namely the constant skin friction −$f''(0)$ and local heat transfer rate −$\theta '(0)$ with the alteration in the control parameters has been discussed by Tables 1 and 2 independently. It is noted that the relaxation time parameter $\beta _1$, number of time retardation $\beta _2$ and magnetic field factor M boosted the coefficient of skin friction coefficient, whereas the Nusselt number −$\theta '(0)$ raise smoothly for higher numerical values of $\beta _2$ and Nt only. Moreover, the number of skin friction enhance as a function of convection parameter $\lambda _c$, but for the remaining parameters both −$f''(0)$ as well as −$\theta '(0)$ have perceived the reverse behaviour. Table 3 presents the verification of the current outcomes for velocity gradient −$f''(0)$ via correlation with already existing paper, and a remarkable acceptance has been gained with those published paper.

**Table 1:** The behavior of − $f''(0)$ where Pr = 0.72, Le = 1, Bi = 2, N = Nt = 0.2, Nb = 0.5, $\delta _t = 0.1$, $\delta _c = 0.3$ are fixed.

$\beta _1$	M	$\lambda$	$\beta _2$	$\gamma$	$\delta$	$\lambda _t$	$\lambda _c$	−$f''(0)$
0.1	0.5	0.1	0.1	0.25	0.25	0.2	0.1	0.6073
0.3								0.7335
0.4								0.7417
0.1	0.3	0.1	0.1	0.25	0.25	0.2	0.1	0.6010
	0.6							0.6084
	0.8							0.7018
0.1	0.5	0.1	0.2	0.25	0.25	0.2	0.1	0.5975
			0.3					0.6017
			0.4					0.6882
0.1	0.5	0.1	0.1	0.25	0.2	0.2	0.1	0.6430
					0.3			0.5486
					0.5			0.4554
0.1	0.5	0.1	0.1	0.25	0.25	0.1	0.1	0.6206
						0.2		0.6073
						0.3		0.5894
0.1	0.5	0.1	0.1	0.25	0.25	0.2	0.2	0.7141
							0.3	0.7281
							0.5	0.7361

**Table 2:** The behavior of − $\theta '(0)$ for $\gamma = \delta = \lambda = \delta _c = \lambda _c = 0.1$, N = 1.0, Nb = 0.3, $\lambda _t =0.2$ are fixed.

$\beta _1$	M	Bi	$\beta _2$	Pr	Le	$\delta _t$	*Nt*	−$\theta '(0)$
0.1	0.5	1.0	0.1	1.0	1.0	0.1	0.1	− 0.4181
0.2								− 0.4188
0.3								− 0.4195
0.1	0.3	1.0	0.1	1.0	1.0	0.1	0.1	− 0.4167
	0.7							− 0.4194
	1.0							− 0.4212
0.1	0.5	0.5	0.1	1.0	1.0	0.1	0.1	− 0.2935
		0.8						− 0.3779
		1.5						− 0.4877
0.1	0.5	1.0	0.2	1.0	1.0	0.1	0.1	− 0.4173
			0.4					− 0.4160
			0.6					− 0.4150
0.1	0.5	1.0	0.1	0.7	1.0	0.1	0.1	− 0.3946
				0.9				− 0.4105
				1.2				− 0.4325
0.1	0.5	1.0	0.1	1.0	0.3	0.1	0.1	− 0.4179
					0.6			− 0.4180
					2.0			− 0.4182
0.1	0.5	1.0	0.1	1.0	1.0	0.2	0.1	− 0.4169
						0.5		− 0.6506
						0.7		− 0.7155
0.1	0.5	1.0	0.1	1.0	1.0	0.1	0.2	− 0.4158
							0.5	− 0.4088
							0.9	− 0.3989

**Table 3:** Result comparison for different values of $\beta _1$.

$\beta _1$	Abel et al.^57^	Megahed^58^	Khan et al.^44^	Current results
0.0	0.999996	0.999978	1.0	1.000429
0.2	1.051948	1.051945	1.0518899	1.051883
0.4	1.101850	1.101848	1.1019033	1.101878
0.6	1.150163	1.150160	1.1501373	1.150162
0.8	1.196692	1.196690	1.1967113	1.196283
1.2	1.285257	1.285253	1.2853632	1.285266
1.6	1.368641	1.368641	1.3687584	1.368241

## Conclusions

In the present work, the coupled mixed convection flow with Oldroyd-B nanofluid through a rotating sphere in the stagnation point is discussed under thermal convective and no mass flux condition. Further, non-Fourier and non-Fick’s mass flux theory is carried out to analysis the features of heat and mass transfer. By means of appropriate non-dimensional analysis the formulated mathematical model has been generated into non-dimensional form, and it is numerically resolved by operating bvp4c method. The major findings in the current analysis were described by means of figures and tables through MATLABR2023a. So, the key outcomes are summarized as:

- All normal velocity, temperature, and concentration were enhanced for constant relaxation time, while the opposite trend have been seen for retardation time parameter.
- For higher magnetic and relaxation time parameters skin friction exhibits increasing behavior, whereas the conflicting tendency is being noted for thermal and solutal convection parameters.
- The expanding thermophoresis parameter watched an enlargement in both temperature and concentration fields. But augmenting in the amount of Brownian motion parameter constant brings down the concentration of nanoparticle.
- As the magnetic field parmeter constant, and rotation variable larger in number the linear velocity marked enhancement, whereas the angular velocity declines corresponding to large magnetic field strength.
- Further, The stream function become dominant for higher values of magnetic field parameter.
- The Nusselt number is enhances for bigger Prandtl number, similarly, same trend is being noticed for augmented Biot number on fluid temperature.
- The rate of heat transfer is declines as a functions of Biot number, while analogous effect is being observed Prandtl number on temperature profile.
- Additionally, for inflated buoyancy force fraction, the distribution of fluid velocity and its bounding line become diminished.
- The upshot also noticed that for an elevating in Lewis number and solutal relaxation time constant the graph of nanoparticle become bigger.
- The increment in rotation parameter, first and second order slip parameter diminishes the skin friction coefficient.

## Data Availability

The data used to support the findings of the study are involved in the article.

## List of symbols

$$B_i$$: Biot number
$B_0$: Magnetic field strength (Tesla)
*C*: Species concentration (mol/m$^3$)
$$c_p$$: Specific heat (J/m^3^ K)
$C_\infty$: Ambient concentration (mol/m^3^)
$C_w$: Wall concentration (mol/m^3^)
$D_B$: Brownian diffusion factor (m^2^/s)
$$D_T$$: Thermophoretic diffusion (m^2^/s)
*f*: Dimensionless stream function
*g*: Dimensionless rotational velocity
*Gr*: Thermal Grashof number
*Gc*: Concentration Grashof number
$h_f$: Convective heat coefficient
*K*: Thermal conductivity (W/mK)
*Le*: Lewis number
*M*: Magnetic parameter
*N*: Buoyancy force ratio
*Nb*: Brownian motion parameters
*Nt*: Thermospheres parameters
*Pr*: Prandtl number
*T*: Fluid temperature (K)
$T_f$: Hot fluid temperature (K)
$$T_\infty$$: Ambient temperature (K)
(*u*, *v*, *w*): Velocity components (m/s)
(*x*, *y*, *z*): Cartesian coordinates (m)

## Greeks

$$\eta$$: Dimensionless variables
$\alpha$: Thermal diffusion coefficient (m^2^/s)
$\beta _1$: Deborah number for relaxation
$\beta _2$: Deborah number for retardation time
$\gamma$: First order slip parameter
$\delta$: Second order slip parameter
$\delta _t$: Thermal time relaxation parameter
$\delta _c$: Concentration time relaxation parameter
$$\lambda$$: Rotation parameter
$$\lambda _1$$: Time relaxation
$$\lambda _2$$: Time retardation
$\lambda _t$: Thermal nonlinear convection
$\lambda _c$: Solutal nonlinear convection
$$\theta$$: Dimensionless temperature
$\Omega$: Angular velocity (rad/s)
$$\phi$$: Dimensionless concentration
$$\nu$$: Kinematic viscosity (m^2^/s)
$\mu$: Dynamic viscosity (kg/ms)
$\kappa$: Vortex viscosity (Pas)
$\rho$: Fluid density (kg/m^3^)
$\tau _{e}$: Relaxation time heat flux
$\tau _c$: Relaxation time mass flux
$$\sigma$$: Electric conductivity (s/m)

## Subscripts

$\infty$: Free stream condition
*w*: Condition at the surface
